# Predicting Intensive Care Unit Admission for COVID-19 Patients from Laboratory Results

**DOI:** 10.1155/2022/4623901

**Published:** 2022-05-26

**Authors:** Basmah M. Azad Allarakia, Hattan S. Gattan, Rawan H. Abdeen, Bassam M. Al-ahmadi, Abdullah F. Shater, Mohammed B. Bazaid, Omar W. Althomali, Abdulrahman S. Bazaid

**Affiliations:** ^1^Medical Department, Althagar General Hospital, Jeddah, Saudi Arabia; ^2^Department of Medical Laboratory Sciences, Faculty of Applied Medical Sciences, King Abdulaziz University, Jeddah, Saudi Arabia; ^3^Special Infectious Agents Unit, King Fahad Medical Research Center, Jeddah, Saudi Arabia; ^4^Diagnostic Radiology Technology Department, Faculty of Applied Medical Sciences, King Abdulaziz University, Jeddah, Saudi Arabia; ^5^Department of Biology, Faculty of Science, Taibah University, Medina, Saudi Arabia; ^6^Department of Medical Laboratory Technology, Faculty of Applied Medical Sciences, University of Tabuk, Tabuk, Saudi Arabia; ^7^Pediatric Department, East Jeddah Hospital, Jeddah, Saudi Arabia; ^8^Department of Physiotherapy, College of Applied Medical Sciences, University of Hail, Ha'il, Saudi Arabia; ^9^Department of Clinical Laboratory Science, College of Applied Medical Science, University of Hail, Ha'il, Saudi Arabia

## Abstract

Trends in routine laboratory tests, such as high white blood cell and low platelet counts, correlate with COVID-19-related intensive care unit (ICU) admissions. Other related biomarkers include elevated troponin, alanine aminotransferase, and aspartate transaminase levels (liver function tests). To this end, the aim of this study was to investigate the effect of changes in laboratory test parameters on ward-based and ICU COVID-19 patients. A total of 280 COVID-19 patients were included in the study and were divided based on admission status into ICU (37) or ward (243) patients. ICU admission correlated significantly with higher levels of several tested parameters, including lactate dehydrogenase, creatinine, D-dimer, creatine kinase, white blood cell count, and neutrophil count. In conclusion, routine laboratory tests offer an indication of which COVID-19 patients are most likely to be admitted to the ICU. These associations can assist healthcare providers in addressing the needs of patients who are at risk of COVID-19 complications.

## 1. Introduction

On 30 January 2020, the World Health Organization (WHO) officially declared the COVID-19 pandemic as a public health emergency of international concern [1, 2]. Symptoms are usually mild, ranging from fever with a dry cough to sore throat, breathlessness, and fatigue [3]. However, vulnerable patients, such as the elderly and those with comorbidities, are at a higher risk of severe complications, including pneumonia, acute respiratory distress syndrome (ARDS), and multiorgan dysfunction [4]. Comorbidities, such as chronic lung disease, severe asthma, cardiovascular disease, diabetes, and compromised immunity, have been associated with hospitalization and higher mortality [5].

During hospitalization, laboratory tests are routinely carried out, and these have generated useful trends. For example, low lymphocyte and eosinophil count, low platelet count, and high white blood cell (WBC) count correlated with severe disease [6]. Other biomarkers that can affect the severity of the disease are elevated troponin, alanine aminotransferase (ALT), and aspartate transaminase (AST) levels (part of liver function tests). Most patients who do not survive COVID-19 had high troponin because of viral myocarditis and cardiac injury, which can then lead to multiple organ failure. Similarly, ALT and AST tend to be elevated in multiple organ failure. Critical changes that occur with organ failure include changes in renal function profiles, blood urea nitrogen (BUN), creatinine, and coagulation markers [7, 8]. Higher D-dimer is also another marker of disease severity and mortality rate found to be elevated in mild to severe cases [5].

These useful trends have been reported in different studies but have not been tested systematically in a hospital setting. Therefore, the aim of the current study was to investigate the link between laboratory test results in a cohort of COVID-19 patients and ICU admission in a hospital in Saudi Arabia.

## 2. Methods

### 2.1. Study Design and Ethics

The work in this report is a retrospective study conducted at East Jeddah General Hospital, Saudi Arabia, from July to 30 August 2020. Data were extracted from medical records of patients who were diagnosed with COVID-19 based on a positive real-time reverse transcriptase polymerase chain reaction (RT-PCR) (Thermo Fisher Scientific, USA) test on nasopharyngeal samples. The test has sensitivity and specificity of 96% and 94%, respectively [9]. The study was approved by the Jeddah Health Affaires ethics board at the Ministry of Health in Saudi Arabia (H-02-J-002-01285). As this is a retrospective study, the requirement for patient consent documentation was waived by the ethics committee.

### 2.2. Data Collection

Patients were categorized into two groups based on admission status to the ICU or isolation ward. Demographic information, admission status, and laboratory results were collected. Test results included complete blood count (CBC), coagulation parameters and platelet count (PLT), activated partial thromboplastin time (APTT), prothrombin time (PT), D-dimer, international normalized ratio (INR), troponin, creatine kinase (CK), and renal function tests (calcium (Ca), sodium (Na), potassium (K), magnesium (Mg), blood urea nitrogen (B) and creatinine, liver function tests total bilirubin (TB), direct bilirubin (DB), aspartate aminotransferase (AST), alanine transaminase (ALT), and lactate dehydrogenase (LDH)).

### 2.3. Statistical Analysis

Data were coded in Excel version 16.57, transferred to SPSS version 27, and analyzed. Before statistical analysis, healthy baseline of all standard test results was set, and each patient was categorized as either having low, normal, or high levels. Descriptive statistical analysis (numbers and percentages) was carried out on demographic characteristics, signs, and symptoms at admission, comorbidities, and number of comorbidities. The categories of each variable (low, normal, high) were compared between patients admitted to the ICU and those in the ward. Chi-square test was used when all the parameters had >5 expected counts; otherwise, Fisher exact test was used. To identify variables testable for correlation, values for the ICU group and the ward group were compared using either a *t*-test when assumptions were met (no outlier and data were normally distributed and homogeneity of variances) or Mann–Whitney when the assumptions were not met. Nine variables including lactate dehydrogenase, creatinine, AST, D-dimer, creatine kinase, white blood cell count, neutrophil count, and eosinophil count were assessed by logistic regression. Statistically significant cut-off was defined at 0.05.

## 3. Results

### 3.1. Demographics and Clinical Features

COVID-19 positive patients (*n* = 280) were included in the study and were divided based on admission status into ICU (*n* = 37) and ward (*n* = 243) patients (Table 1). The participants were predominantly male in both groups (84% and 76% for ICU and non-ICU, respectively), and the majority of patients were from Yemen, Saudi Arabia, and Bangladesh. Just over half of those admitted to the ICU (53%) were over 50 years old, while 40% of ward patients were younger (<40 years old).

Cough, fever, and shortness of breath were the most common signs reported at admission among ward and ICU patients, followed by diarrhea and hypoxia, respectively. Over half of the patients did not have underlying conditions, while 1 in 7 and 1 in 9 of the remaining group had diabetes or hypertension, respectively. Approximately 16% of those admitted to the ICU had two comorbidities compared with 6% of those admitted to the isolation ward (Table 1).

### 3.2. Laboratory Test Results

The percentage of patients with low, normal, and high levels (Supplement 1) of blood urea nitrogen, creatinine, prothrombin time, troponin, creatine kinase, white blood cell count, neutrophil count, lymphocyte count, and eosinophil count was significantly different (*p* < 0.05) between ward and ICU patients (Table 2 and Supplement 2). A high percentage of patients with high (abnormal) levels of blood urea nitrogen, creatinine, creatine kinase, and white blood cell count were observed with the ICU group compared with ward patients. In contrast, a high percentage of patients with low (abnormal) and a low percentage of patients with normal lymphocyte count and eosinophil count were observed in the ICU group compared with ward patients. The percentage of ICU patients with a normal prothrombin time was significantly lower than the ward group, while the ICU group showed a higher percentage of patients with high troponin levels compared with ward patients (Table 2).

### 3.3. Association between Admission Status and Laboratory Test Results

A binomial logistic regression was performed to ascertain the effect of changes in laboratory results on the likelihood that a patient is admitted to the ICU. The outcome of logistic regression was statistically significant (*p* < 0.005). The model explained 62% (Nagelkerke *R*^2^) of the variance associated with ICU admission and correctly classified 92.5% of ICU cases. Of the abnormal laboratory tests, six predictor variables were statistically significant: lactate dehydrogenase, creatinine, D-dimer, creatine kinase, white blood cell count, and neutrophil count (Table 3). Patient with an increase in white blood cells by 1.07 × 10^9^/L had 2.9 times higher odds to be admitted to the ICU. Those with low creatinine, D-dimer, creatine kinase, and neutrophil count were associated with a reduction in the likelihood of ICU admission.

## 4. Discussion

Several important factors have been reported to affect the outcome of COVID-19, including age, immune state, comorbidities, sex, and health services [10, 11]. In a pandemic outbreak, triage and patient stratification may help in managing and diagnosing critical cases earlier [11]. Patients with COVID-19 have different types of symptoms, ranging from mild to severe, which can be important in triage. In line with previous studies, the most prevalent symptoms in the patient cohort in this study included fever, cough, fatigue, shortness of breath, and dyspnoea [12, 13]. In addition, COVID-19 patients who were male, at least 55 years old or had a comorbidity (diabetes mellitus or hypertension), had increased risk of ICU admission. Similar correlations between these factors and ICU admission have been reported [5, 14–16]. To date, there is no clear explanation why age is an independent risk factor. One hypothesis is related to changes that occur in the anatomy of the lung and muscle atrophy, which affect the physiological function of the respiratory system [17]. Changes to the composition of the immune system are another explanation for age-related risks, leading to a decrease in the ability to respond to infection [18]. Furthermore, a cytokine storm occurs at later stages of infection, causing inflammation in major organs, such as the lungs, heart, brain, liver, and kidneys, which aggravates dyspnoea and hypoxemia [19]. Being male was associated with higher risk of ICU admission [20–22]. Research has demonstrated that the X chromosome and sex hormones play a significant role in immune response [23]. Male sex hormones regulate angiotensin-converting enzyme 2 (ACE2), making males more susceptible to SARS-CoV-2 infection and leading to poor prognosis [24, 25].

Comorbidities, such as hypertension and diabetes, have been reported previously to affect the severity of the disease and the prognosis of COVID-19 patients [14, 26–28]. Several mechanisms have been discussed for the effect of hypertension, one of which is related to distribution of ACE2 enzyme/receptor, expressed in different tissues, such as lung alveolar epithelial cells, cardiovascular, arterial smooth muscle, arterial and venous endothelial cells, and enterocytes of the small intestine [29]. SARS-CoV-2 enters human cells through interaction with ACE2 [30], with reduced or abolished ACE2 expression reported to prevent virus entry to alveolar epithelial cells and to lead to vasoconstriction [30]. In line with our findings, a national study conducted in Saudi Arabia reported that the two most common comorbidities in COVID-19 patients were diabetes and hypertension [31]. However, these are in contrast to another study which found a lack of association between diabetes and COVID-19 severity [32]. This highlights variation between different populations and the importance of national surveillance studies.

Several studies investigated the link between laboratory results and admission to the ICU in different population. There was a significant reduction in lymphocyte, monocyte, eosinophil, and platelet counts as well as hemoglobin levels in severe cases compared with mild and moderate cases [33]. The study also highlighted the higher neutrophil count, ALT, AST, creatinine, and BUN in COVID-19 patients with severe symptoms and possible admission to the ICU [3, 34]. Other biomarkers, such as LDH, PT, and D-dimer, were also associated with the severity of the disease and admission to the ICU [33–35]. Moreover, a report has found that patients with elevated leucocyte count tend to have more severe symptoms with possible admission to the ICU compared with nonsevere cases [36]. The study also found that ICU patients will have higher D-dimer and PT than nonsevere cases. It was reported that several laboratory tests, such as WBC count, platelet count, and C-reactive protein (CRP), are helpful to predict whether COVID-19 patients are likely to be admitted to the ICU [35, 37]. Patients with a high WBC count and CRP are at a higher risk of severe disease and being admitted to the ICU [37, 38]. Our results indicate an association between elevated white blood cell and neutrophil counts with the severity of the disease and admission to the ICU.

The highlighted studies investigated a range of laboratory tests in different populations, with trends mostly in line with our findings, and we performed further analysis in a Saudi hospital setting to ascertain the link of laboratory tests and the likelihood of admission to the ICU [39], although few studies investigate admission predictors to ICU among COVID-19 in Saudi Arabia. Furthermore, high lactate dehydrogenase is used as a biomarker for acute respiratory distress syndrome (ARDS) and death in severe COVID-19, where lactate dehydrogenase is secreted into the circulation in response to the infection [40]. In addition, patient with low creatinine and creatine kinase levels had reduced risk of admission to the ICU [41]. Creatine kinase is an indicator of muscle damage, and high levels are associated with poor outcome for COVID-19 severe cases [41]. Elevated creatine kinase at admission was associated with those who experienced severe outcomes and had unfavorable prognosis. Critically ill COVID-19 patients are more susceptible to acute kidney injury and increased serum creatinine [42].

In line with our findings, increased serum D-dimer is a common feature of severe COVID-19 infection and has been strongly associated with mortality [5]. D-dimer is a key diagnostic biomarker for thrombotic disorders, with an inconclusive link to pneumonia. Research on COVID-19 patients has recently highlighted the association between elevated D-dimer and thrombotic complications among severe COVID-19 cases [43] . Our findings highlight that D-dimer levels are higher in patients admitted to the ICU compared with those admitted to the isolation ward. Similarly, a previous study demonstrated the use of D-dimer as a potential early marker for predicting in-hospital mortality in 343 patients in China, with a cut-off of 2 *μ*g/ml.

## 5. Conclusion

The findings in this study suggest that different laboratory test results can serve as predictors of the likelihood of admission of COVID-19 patients to the ICU. These laboratory biomarkers include lactate dehydrogenase, creatinine, D-dimer, creatine kinase, white blood cell count, and neutrophil count. Elevations of these biomarkers indicate increased risk of being admitted to the ICU.

## Figures and Tables

**Table 1:** Demographics and clinical features of patients admitted to the ICU and isolation ward.

	ICU	Ward	Total
Age (years), mean (SD)	55.35 (13)	45.98 (14)	47.21 (15)
Age range			
<40	5 (13.5)	97 (39.9)	102
40–49	6 (16.2)	51 (21.0)	57
50–59	12 (32.4)	50 (20.6)	62
60–69	8 (21.6)	26 (10.7)	34
70–79	3 (8.1)	14 (5.8)	17
≥80	3 (8.1)	5 (2.1)	8

Sex			
Male	31 (83.8)	184 (75.7)	215
Female	6 (16.2)	59 (24.3)	65

Nationality			
Saudi Arabia	5 (13.5)	58 (23.9)	63
Yemen	8 (21.6)	59 (24.3)	67
Egypt	2 (5.4)	20 (8.2)	22
Palestine	2 (5.4)	11 (4.5)	13
Syria	0	5 (2.1)	5
Sudan	1 (2.7)	10 (4.1)	11
Bangladesh	8 (21.6)	29 (11.9)	37
Pakistan	5 (13.5)	12 (4.9)	17
India	2 (5.4)	11 (4.5)	13
Afghanistan	0	6 (2.5)	6
Burma	1 (2.7)	8 (3.3)	9
Philippines	2 (5.4)	4 (1.6)	6
Other	2 (5.4)	10 (4.1)	12

Signs and symptoms at admission			
Asymptomatic	1 (1.2)	18 (1.6)	19
Fever	22 (27.2)	167 (15.0)	189
Shortness of breath	21 (25.9)	116 (10.4)	137
Cough	19 (23.5)	164 (14.7)	183
Diarrhea	1 (1.2)	28 (2.5)	29
Vomiting	1 (1.2)	11 (1.0)	12
Chest pain	2 (2.5)	11 (1.0)	13
Abdominal pain	2 (2.5)	2 (0.2)	4
Loss of apatite	0	5 (0.4)	5
Loss of smell and taste	0	2 (0.2)	2
Respiratory distress syndrome	2 (2.5)	0	2
Sore throated	0	5 (0.4)	5
Fatigue	2 (2.5)	9 (0.8)	11
Headache	2 (2.5)	22 (2.0)	24
Hypoxia	3 (3.7)	0	3
Nausea	0	8 (0.7)	8
Other	2 (2.5)	4 (0.1)	6

Comorbidities			
No underlying conditions	24 (55.8)	160 (60.6)	184
Hypertension	5 (11.6)	31 (10.9)	36
Diabetes mellitus (DM)	7 (14.0)	39 (13.4)	46
Asthma	0	3 (1.1)	3
Hypothyroidism	0	5 (1.8)	5
Dyslipidemia	0	6 (2.1)	6
Heart disease	0	5 (1.8)	5
Kidney disease	0	4 (1.4)	4
Breast cancer	0	2 (0.7)	2
Smoker	2 (2.3)	2 (0.7)	4
Other	3	7 (0.4)	10

Number of comorbidities			
None	24 (55.8)	160 (60.6)	184
One	7 (18.9)	55 (22.6)	62
Two	6 (16.2)	15 (6.2)	21
Three or more	0	13 (5.3)	13

**Table 2:** The number and percentage of patients (admitted to the ICU or isolation ward) with normal or abnormal (low/high) laboratory test results.

Test		ICU		Ward		*p* value	*z*-comparison
Sodium	Low	26	70.3%	148	60.9%		
	Normal	10	27.0%	95	39.1%	0.053	
	High	1	2.7%	0	0.0%		

Potassium	Low	6	16.2%	13	5.3%		
	Normal	29	78.4%	213	87.7%	0.068	
	High	2	5.4%	17	7.0%		

Lactate dehydrogenase	Low	0	0.0%	1	0.4%		
	Normal	1	2.7%	11	4.5%	0.9	
	High	36	97.3%	231	95.1%		

Blood urea nitrogen	Low	10	27.0%	55	22.6%		
	Normal	20	54.1%	174^∗^	71.6%	*0.01*	*0.036*
	High	7	18.9%	14^∗^	5.8%		*0.012*

Creatinine	Low	0	0.0%	4	1.6%		
	Normal	19	51.4%	231^∗∗^	95.1%	*<0.01*	*<0.01*
	High	18	48.6%	8^∗∗^	3.3%		*<0.01*

Aspartate aminotransferase	Low	0	0.0%	5	2.1%		
	Normal	11	29.7%	70	28.8%	0.5	
	High	26	70.3%	168	69.1%		

Alanine transaminase	Low	3	8.1%	22	9.1%		
	Normal	28	75.7%	197	81.1%	0.48	
	High	6	16.2%	24	9.9%		

Activated partial thromboplastin time	Low	7	18.9%	36	14.8%		
	Normal	26	70.3%	182	74.9%	0.73	
	High	4	10.8%	25	10.3%		

Prothrombin time	Low	8	21.6%	27	11.1%		
	Normal	23	62.2%	202^∗^	83.1%	*0.01*	*0.006*
	High	6	16.2%	14	5.8%		

International normalized ratio	Low	1	2.7%	2	0.8%		
	Normal	25	67.6%	138	56.8%	0.16	
	High	11	29.7%	103	42.4%		

D-dimer	Low	0	0.0%	14	5.8%		
	Normal	9	24.3%	64	26.3%	0.38	
	High	28	75.7%	165	67.9%		

Troponin	Low	4	10.8%	55	22.6%		
	Normal	30	81.1%	187	77.0%	*<0.01*	
	High	3	8.1%	1^∗^	0.4%		*0.008*

Creatine kinase	Low	1	2.7%	10	4.1%		
	Normal	8	21.6%	211^∗∗^	86.8%	*<0.01*	*<0.01*
	High	28	75.7%	22^∗∗^	9.1%		*<0.01*

White blood cell count	Low	0	0.0%	23	9.5%		
	Normal	22	59.5%	192^∗^	79.0%	*<0.01*	*0.013*
	High	15	40.5%	28^∗∗^	11.5%		*<0.01*

Neutrophil count	Low	1	2.7%	36^∗^	14.8%		*0.039*
	Normal	21	56.8%	179	73.7%	*<0.01*	
	High	15	40.5%	28^∗∗^	11.5%		*<0.01*

Lymphocyte count	Low	32	86.5%	135^∗∗^	55.6%		*<0.01*
	Normal	5	13.5%	103^∗∗^	42.4%	*<0.01*	*<0.01*
	High	0	0.0%	5	2.1%		

Monocyte count	Low	1	2.7%	27	11.1%		
	Normal	32	86.5%	194	79.8%	0.25	
	High	4	10.8%	22	9.1%		

Eosinophil count	Low	30	81.1%	141^∗^	58.0%		*0.007*
	Normal	7	18.9%	98^∗^	40.3%	*0.03*	*0.017*
	High	0	0.0%	4	1.6%		

Basophil count	Low	19	51.4%	164	67.5%		
	Normal	17	45.9%	66	27.2%	0.07	
	High	1	2.7%	13	5.3%		

Hemoglobin	Low	7	18.9%	56	23.0%		
	Normal	27	73.0%	154	63.4%	0.56	
	High	3	8.1%	33	13.6%		

Platelet count	Low	10	27.0%	50	20.6%		
	Normal	25	67.6%	179	73.7%	0.62	
	High	2	5.4%	14	5.8%		

^∗^ *p* < 0.05, ^∗∗^*p* < 0.01. Italic indicates significant difference compared with those admitted to the ICU. Normal ranges are in Supplement 1.

**Table 3:** Logistic regression to assess associations between abnormal laboratory tests and admission to the ICU.

	*B*	SE	*p* value	Odd ratio	Exp (*B*)
					95% CI
Lactate dehydrogenase	-0.006	0.003	0.036	0.994	0.989
Creatinine	-2.561	0.809	0.002	0.077	0.016
D-dimer	-0.312	0.143	0.029	0.732	0.553
Creatine kinase	-0.007	0.001	0.0001	0.993	0.990
White blood cell count	1.074	0.417	0.010	2.928	1.293
Neutrophil count	-1.420	0.452	0.002	0.242	0.100
Eosinophil count	-2.458	1.290	0.057	0.086	0.007
Constant	9.520	1.678	0.0001		

*B*: log odd; S.E: standard error; 95% CI: 95% confidence interval.

## Data Availability

The datasets used and/or analyzed during the current study are available within the manuscript.

## Supplementary Materials

Supplementary Materials Supplements 1: low, normal, and high range for laboratory tests included in the study. Supplement 2: median of laboratory results among ICU and ward patients.
