# Supplementary material for: Predicting Intensive Care Unit Admission for COVID-19 Patients from Laboratory Results
Source: Dis Markers. 2022 May 26;2022:4623901. doi: 10.1155/2022/4623901 (PMC9133894; doi:10.1155/2022/4623901)
Supplement: Supplementary Materials — Supplements 1: low, normal, and high range for laboratory tests included in the study. Supplement 2: median of laboratory results among ICU and ward patients. [file 4623901.f1.docx]

**Supplements 1: Low, normal and high range for laboratory tests included in the study**

| **Test** | **Low** | **Normal range** | **High** |
| --- | --- | --- | --- |
| Sodium | ≤136 mmol/L | 136.1-145 mmol/L | >145 mmol/L |
| Potassium | ≤3.5 mmol/L | 3.6-5.1 mmol/L | >5.1 mmol/L |
| Lactate dehydrogenase | ≤100 u/L | 100.1-190 u/L | >190 u/L |
| Blood urea nitrogen | ≤ 7mg/dL | 7.1-20 mg/dL | > 20 mg/dL |
| Creatinine | ≤0.55 mg/dL | 0.56-1.3 mg/dL | >1.3 mg/dL |
| Aspartate aminotransferase | ≤15 u/L | 15.1-37 u/L | >37 u/L |
| Alanine transaminase | ≤12 u/L | 12.1-78 u/L | >78 u/L |
| Activated partial thromboplastin time | ≤ 26 sec | 26.1-40 sec | Above 40 sec |
| Prothrombin time | ≤ 10 sec | 10.1-14.5 sec | Above 14.5 sec |
| International normalized ratio | ≤ 0.86 sec | 0.87-1.3 sec | Above 1.3 sec |
| D-dimer | 0 | 0.01-0.5 | Above 0.5 |
| Troponin | 0 ng/L | 0.01-350 ng/L | >350 ng/L |
| Creatine kinase | ≤26 u/L | 26.1-308 u/L | >308 u/L |
| White blood cell count | ≤3.5 ×10^9^/L | 3.6–9.5×10^9^/L | >9.5 ×10^9^/L |
| Neutrophil count | ≤2 ×10^9^/L | 2.1–7 ×10^9^/L | >7 ×10^9^/L |
| Lymphocyte count | ≤1.5 ×10^9^/L | 1.5–4 ×10^9^/L | >4 ×10^9^/L |
| Monocyte count | ≤0.2×10^9^/L | 0.2–1 ×10^9^/L | >1 ×10^9^/L |
| Eosinophil count | ≤ 0.02 ×10^9^/L | 0.02–0.5 ×10^9^/L | >0.5 ×10^9^/L |
| Basophil count | ≤0.01 ×10^9^/L | 0.01–0.1 ×10^9^/L | >0.1 ×10^9^/L |
| Haemoglobin | ≤12 g/dL | 12-15 g/dL | >15 g/dL |
| Platelet count | ≤150 ×10^9^/L | 150–450 ×10^9^/L | >450 ×10^9^/L |

.


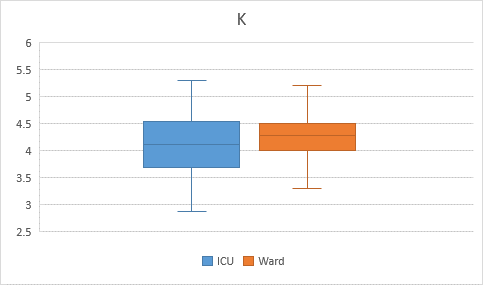

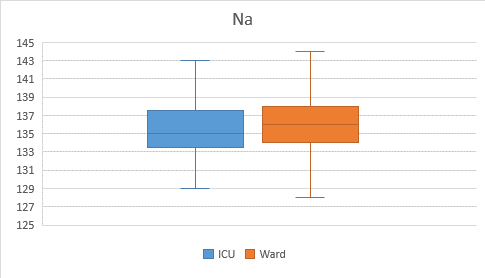

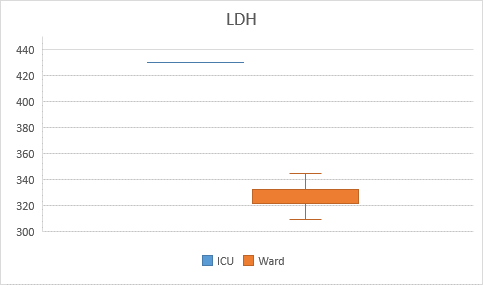

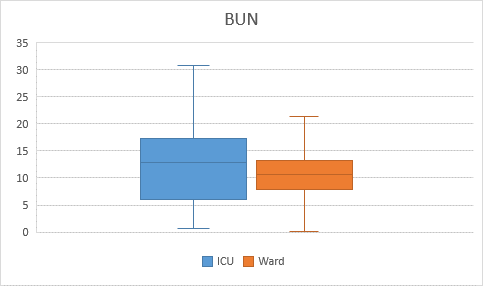


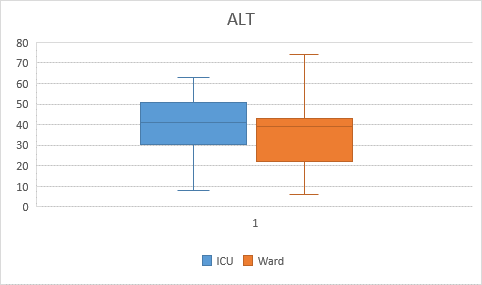

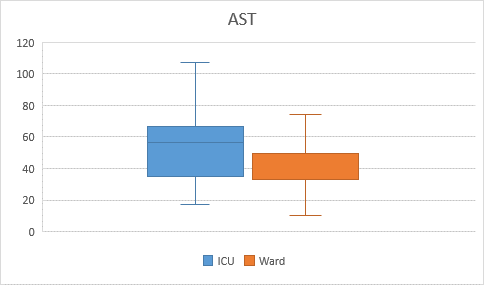

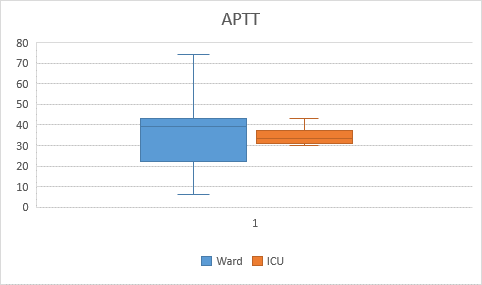

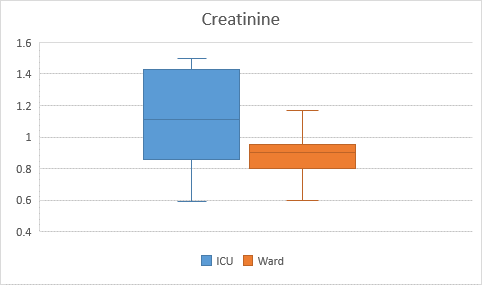

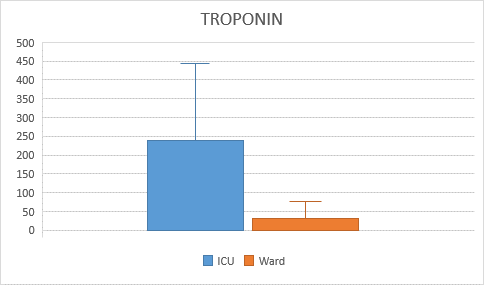

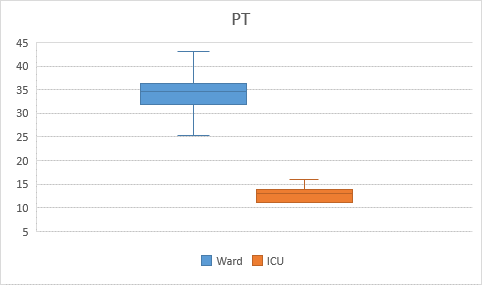

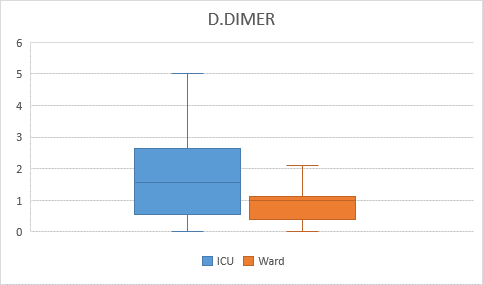

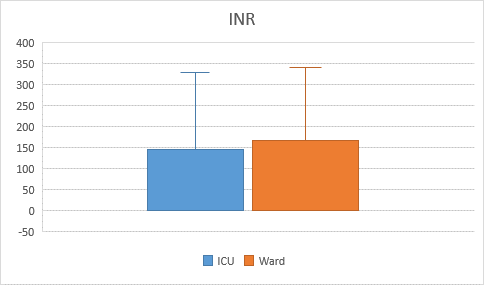

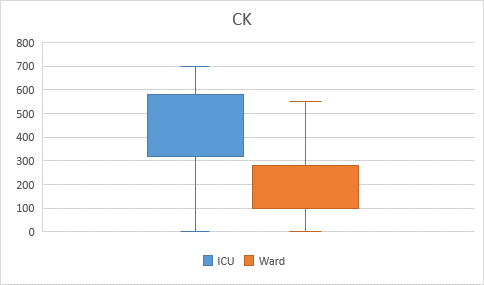

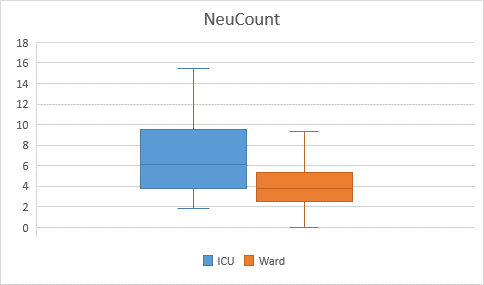

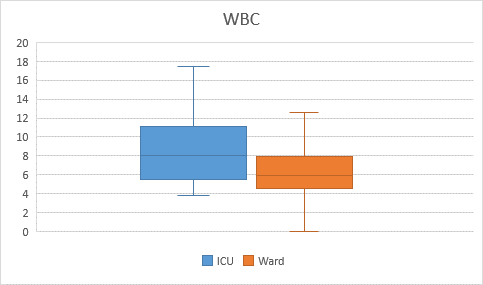

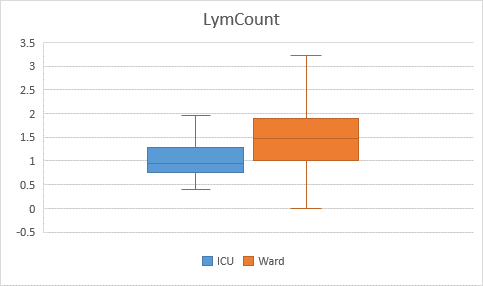

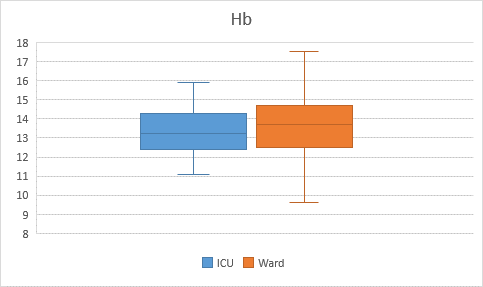

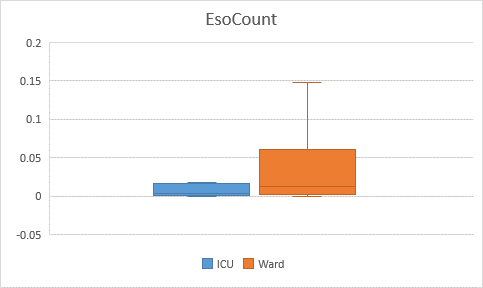

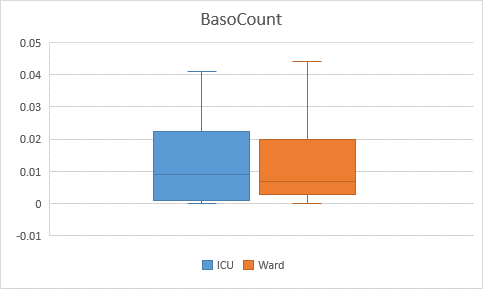

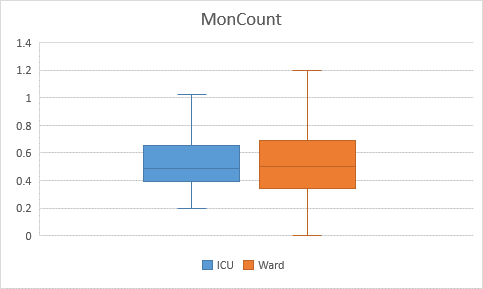

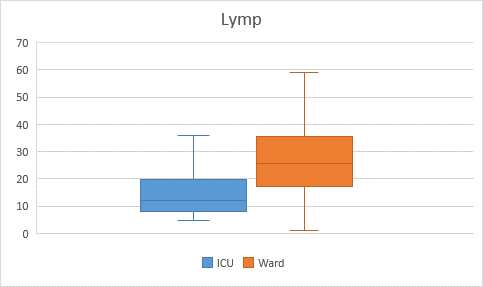

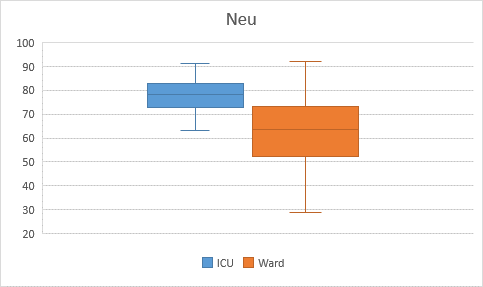

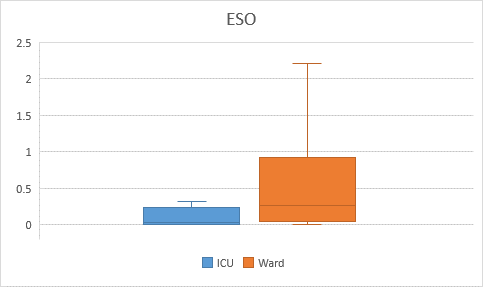

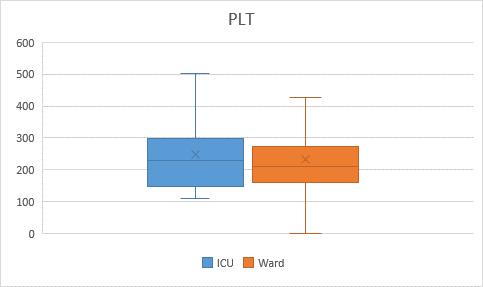

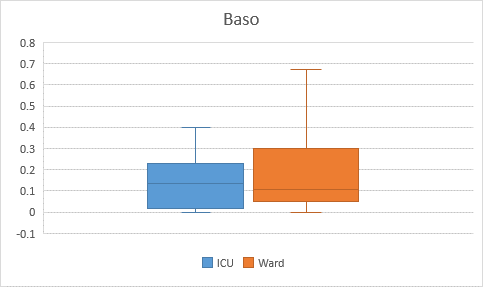

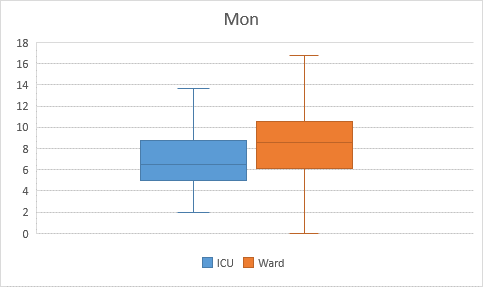


**Supplement 2: Median of laboratory results among ICU and ward patients**
